# Improving Knowledge and Attitudes About Child Trauma Among Parents and Staff in Head Start Programs

**DOI:** 10.1007/s10995-022-03473-8

**Published:** 2022-08-24

**Authors:** A. Guerrero, A. Herman, C. Teutsch, R. Dudovitz

**Affiliations:** 1 Department of Pediatrics and Children’s Discovery and Innovations Institute, David Geffen School of Medicine, UCLA Mattel Children’s Hospital, 10833 LeConte Ave. 12-358 CHS, Los Angeles, CA 90095 USA; 2 UCLA Health Care Institute, Anderson School of Management, University of California Los Angeles, 110 Westwood Plaza, Los Angeles, CA 90095 USA

**Keywords:** Early childhood, Trauma, Early care and education, Health literacy, train-the-trainer

## Abstract

**Background:**

Early childhood represents a sensitive developmental period when trauma-informed care may mitigate the effects of trauma on developmental and health outcomes. However, few interventions use a low-literacy scalable approach to improve child trauma knowledge and attitudes among parents and early childcare and education caregivers.

**Methods:**

Representatives from 24 early head start (EHS) and head start (HS) agencies attended a 2 day online train-the trainer session and then delivered a child trauma and resilience training to staff at their sites, with the option to deliver a similar training to parents. Baseline and 3 month post-training surveys assessed participant knowledge and attitudes regarding childhood trauma and resilience. Paired T-tests and chi2 analyses assessed changes in responses over time.

**Results:**

Thousand five hundred sixty seven staff from 24 agencies and 443 parents from 7 agencies completed baseline and follow up surveys. Over 55% of parents reported their child had experienced at least one adverse childhood experience. Staff and parents had high knowledge regarding causes of trauma at baseline. Both staff and parents, demonstrated significant improvements in identifying symptoms of child trauma. Staff also improved knowledge of resiliency and toxic stress. Parents reported more positive attitudes towards trauma-informed parenting practices.

**Conclusion:**

This is the first training on childhood trauma among EHS/HS providers and parents using a low literacy train-the-trainer approach. Results suggest a potentially promising methodology with broad dissemination potential to prepare and train the one million plus teachers and caregivers in center-based settings and the parents and families who access them to recognize and respond to child trauma.

**Supplementary Information:**

The online version contains supplementary material available at 10.1007/s10995-022-03473-8.

## Significance

Although trauma-informed care has the potential to mitigate the harmful effects of adverse childhood experiences on health, few successful strategies exist to enhance the ability of early childhood parents and providers to understand, recognize, and respond to child trauma. This virtual train-the-trainer program successfully improved trauma-related knowledge and attitudes among a large and diverse set of participants and could be a promising approach to building the capacity of the early care and education community to be trauma-informed.

## Introduction

Early and cumulative exposure to trauma during sensitive periods of child’s development is associated with life long chronic health conditions like depression, asthma, heart disease, and obesity (Anda et al., 2006; Ports et al., 2019; Su et al., 2015). Studies document short and longer-term consequences of child trauma on childhood adolescent, and early adult outcomes, as well as intergenerational impacts (Bucci et al., 2016; Burke et al., 2011; McKelvey et al., 2019; Thakur et al., 2020). Hence, addressing childhood trauma has emerged as a public health priority (Hughes et al., 2017).

Children exposed to trauma are not destined to poor physical and mental health outcomes if early recognition and supportive, trauma-informed strategies are provided. This is particularly relevant for parents of young children and caregivers in early care and education settings (ECEs). Early childhood represents a sensitive developmental period when trauma-informed care may mitigate the effects of trauma on brain development and neurophysiology and support children in developing resilience and secure attachment relationships with their caregivers (Garner & Shonkoff, 2012; Traub & Boynton-Jarrett, 2017). It is critical for parents of young children and ECE caregivers to understand and recognize common behavioral and health needs of young children who have experienced trauma. An increase in this knowledge can shift attitudes about how parents and ECE caregivers trespond to children’s needs. This is an important first step to ultimately change parenting and caregiver behaviors to better align with trauma informed care (Traub & Boynton-Jarrett, 2017).

The ability to intervene, however, is dependent on the knowledge and understanding of child trauma. Risk factors for childhood trauma, poor access to positive supports, and low health literacy are overlapping (Goldstein et al., 2020), making it particularly important to develop programs that support trauma-informed parenting and early care practices for low-literacy populations. However, few interventions use a low-literacy scalable approach to improve the knowledge and understanding of child trauma among parents and ECE caregivers. The health care institute (HCI), in collaboration with the National Center for Early Childhood Health and Wellness, used a low-literacy train-the-trainer (TTT) approach to train front line staff and parents of children attending early head start (EHS) and head start (HS) agencies from diverse communities across the U.S. The focus of this manuscript is to describe the TTT training and the effectiveness of the training in improving knowledge and attitudes regarding child trauma and effective responses to trauma among EHS/HS staff and parents.

## Methods

### Program Description

The health care institute (HCI) uses a strategic and systematic approach to deliver health promotion training programs for EHS/HS agencies across the US (Sivanand et al., 2017), focusing on increasing leadership skills, building management and workforce capacity, implementation strategies for EHS/HS agencies to work effectively with parents, staff, and community stakeholders, and using low health literacy materials for health promotion. Trainings have addressed common childhood health conditions including oral health, child safety, managing common childhood illnesses, and nutrition and physical activity practices (Herman et al., 2013). Previous evaluations of the oral health and nutrition and physical activity trainings demonstrated improvements in parental knowledge, attitudes, self-reported health behaviors, and health outcomes (Dudovitz et al., 2020; Herman et al., 2012). In 2019, the HCI developed a new training focused on child trauma.

Through a national application process 28 EHS/HS agencies were selected and invited to commit to a four-year program focused on health promotion and family engagement. Agencies were selected based on a total score from their statements accompanying the application with the following criteria: (1) what current programs or initiatives are in place to address health needs of staff, children, and families; (2) successes and barriers that have been encountered with prior initiatives; and (3) description of how participation in the training will benefit their children, families, and community. Each year, a team from the EHS/HS agency, attended a two-day training to build specific knowledge and skills on a health topic so that each agency could: (1) develop a coordinated and strategic plan to promote health; (2) learn and apply implementation strategies to engage staff, parents, children, and community stakeholders around a health topic; (3) learn and apply adult learning principles and delivery techniques to teach health promotion; and (4) deliver consistent dynamic, low health literacy health content to staff and parents.

During the trainings, a systematic approach was used to build leadership and workforce capacity using a TTT model around four child health topics. Sessions included: (1) strategies to increase motivation and buy-in; (2) a roadmap to guide goals, planning, and action steps; (3) a budget plan; and (4) modeling a mock session using power point slides, handouts, and examples of hands-on-activities demonstrating effective implementation of parent training. In the third year, all agencies complete an on-line TTT event, in lieu of in-person training, on four mental health topics: “Early Childhood Trauma and Adversity,” “Positive Discipline,” “Understanding Stress,” and “Understanding Depression.” In March 2019, four webinar sessions (90 min each) over two days were held, following the same approaches used in the in-person two-day TTT events in prior years. The first day of the training focused on all of these topics with *staff as the intended audience*, and the second day with *parents as the intended audience*. All EHS/HS agencies received the training on trauma for staff and then each agency selected two of the four mental health topics for parents. At the conclusion of the training a satisfaction survey was administered to the EHS/HS participants which showed favorable feedback (82% indicated high satisfaction with the webinars, 86% reported improved knowledge on the subjects, and 91% were satisfied with the content expertise of the presenters).

In order to support the EHS/HS agencies with consistent content and messages, “ready-to-use” slides, a four-page brochure, and handouts were provided focusing on: (1) defining trauma and resilience; (2) learning the consequences of trauma in early childhood; (3) identifying the signs and symptoms of trauma; and (4) practical strategies to build resilience. Low-literacy content and materials were co-developed by Georgetown University’s center for child and human development (GUCCHD) in partnership with HCI, under the auspices of the National center on early childhood health and wellness (NCECHW). Using the provided materials each EHS/HS team was then expected to conduct a 90 min training first for at least 50 staff and then, if the agency chose this topic from the menu of mental health topics for parent education, 50 parents from their respective sites. Each agency had the flexibility to determine when the trainings would be delivered and what reinforcement activities would be used for both staff and parents. EHS teams were only required to conduct at least two home visits and use of the tip sheets provided during the training (see Supplementary material) to reinforce the content. All programs were provided with door prizes related to wellness such as: Yoga mats, meditation videos, stress balls, essential oils and aromatherapy bath and body works in order to encourage parent participation*.* Flexibility on the implementation strategies was intentional in order for each agency to best meet local needs and cultures while aligning the activities with required federal Head Start performance standards.

Each team trained staff during August 2019–October 2019 and those sites that chose to deliver the trauma training to parents, trained parents November 2019–January 2020. Activities to reinforce the training occurred September 2019–November 2019 and December 2019–March 2020, for staff and parents, respectively.

### Data Collection

Separate surveys for parents and staff measured knowledge and attitudes. Surveys were developed by GUCCHD and the HCI and designed for a 6th-grade reading level. Surveys were made available in English and Spanish. Baseline surveys were completed immediately before starting a staff or parent training. Follow up surveys were conducted approximately three months later, following reinforcement activities. Participant names were used to link baseline to follow-up surveys. This study was reviewed and determined to be exempt by the **** Institutional Review Board.

### Measures

#### EHS/HS Staff Knowledge

Seven true or false statements assessed staff knowledge regarding trauma and resilience, for example, “Resilience is recovering from or adjusting to misfortune or change; the ability to ‘bounce back’ or overcome odds.” Responses were scored 0 or 1 based on an incorrect or correct response. To assess knowledge regarding signs and symptoms of trauma in young children, staff were asked to mark all that apply from a list of seven behaviors such as fearfulness, eating and sleeping problems, and aggressiveness. Finally, staff were asked three multiple choice questions about adverse childhood experiences (ACEs) and Trauma Informed Care: the definition of ACEs; components of ACEs training; and principles of trauma informed care. Each correct answer was scored as 1, and an incorrect answer as 0.

#### EHS/HS Parent Knowledge, Attitudes, and Experiences with Trauma

Three items assessed knowledge about trauma and resilience. Parents were asked to respond “yes” or “no” to, “Have you heard of adverse childhood experiences (ACEs)?” In addition, parents were asked to indicate “true” or “false” to the statements, “Nurturing and responsive care can help a child who has experienced trauma” and “It is important to talk to young children about things that happened and help them understand the experience.” Responses were scored 1 if a “yes” was marked about an awareness of ACEs or “true” to a correct statement about resiliency. To assess knowledge related to possible causes of trauma, parents were asked to mark all that apply from a list of life events such as, “Being neglected or not cared for.” To assess knowledge regarding signs and symptoms of trauma in young children, parents were asked to mark all that apply from a list of seven behaviors such as fearfulness, eating and sleeping problems, and aggressiveness.

Parental attitudes towards promoting resilience was measured using a three Likert response option to eight statements. For example, parents indicated whether they agree, somewhat agree, or disagree with the following statement, “I can support my child by being calm and patient.” Responses ranged from 1 to 3, with higher scores indicating stronger agreement. Parents were also asked to indicate how much they "believe there are things you can do to help your child if he or she has experienced trauma" with four response categories: “nothing”, “a little”, “a fair amount”, and “a lot.” This item was scored 1–4 with higher scores indicating a greater ability to support a child who has experienced trauma. Parents were asked whether their child had been exposed to adverse childhood experiences, with response options of “yes,” “no,” and “don’t know.” Finally, parents were asked, “In your child’s life, has she/he experienced any of the following?” with potential answers including: (1) violence, abuse or neglect; (2) death or separation from a primary caregiver; (3) parent substance abuse or untreated mental illness; and (4) homelessness and/or lack of food. Responses were coded as 0–4, corresponding to the number of items selected from the list.

### Data Analysis

Data were analyzed separately for staff and parents. Follow up data was missing from four agencies either because a participating agency experienced lead staff turnover or changed leadership. Descriptive statistics were generated for all variables. Differences in baseline values were examined for those with and without follow up data using. Among those with both baseline and follow up surveys, paired t-tests for variables with continuous outcomes and Chi square tests for variables with categorical responses were completed to evaluate for differences in baseline and post-training assessments. Subanalyses by site explored whether changes in pre and post parent responses varied by site. All analyses were conducted in STATA (version 15, StataCorp).

## Results

### Staff

In total, 1567 staff completed baseline and follow up assessments across 24 agencies. The majority of staff at the 24 participating agencies had a Bacherlors’ degree or a higher level of education (72%) followed by an Associate degree (23%) and lastly a Child Development Associate Credential (5%). As seen in Table 1, 84% of baseline participants completed the follow up assessment. Those lost to follow up were slightly less likely than those with follow up data to identify anxious behavior as a possible sign of child trauma, although both groups had high knowledge in this area (96% vs. 99%). There were no other significant differences in baseline assessments between staff with and without follow up data. At baseline, virtually all staff were knowledgeable regarding the definition of trauma, but fewer were able to correctly identify the definitions of resilience and toxic stress (46% and 66%, respectively). Nearly all staff identified avoidant and anxious behavior, as well as irritability as potential symptoms or signs of child trauma but fewer identified being fearful (67%), having eating or sleeping problems (60%), loss of skills/regression (59%), and aggressive behaviors (62%) as potential signs. Lastly, approximately one quarter of staff were not familiar with the principles of trauma informed care.

**Table 1:** Baseline statistics for staff with and without follow up data

	With follow up (N = 1567)	Without follow up (N = 293)	P-value
Knowledge of child trauma			
Definition of trauma	99.6%	99.6%	0.91
Definition of resilience	46.1%	51.5%	0.12
Child trauma	62.8%	63.8%	0.73
Trauma, disease and health	97.4%	98.9%	0.19
Definition of toxic stress	66.3%	72.3%	0.07
Symptoms of child trauma	80.6%	80.4%	0.75
Trauma in early childhood	95.2%	95.2%	0.87
Knowledge of symptoms and signs of child trauma			
Avoidant	91.1%	94.5%	0.05
Anxious	98.6%	95.9%	**0.02**
Fearful	66.8%	65.5%	0.66
Eating and Sleeping Problems	60.1%	62.5%	0.63
Irritable	94.1%	92.1%	0.21
Loss of Skills	59.4%	57.7%	0.50
Aggressive	61.7%	61.8%	0.97
Total # of symptoms identified	5.3	5.3	0.99
Knowledge of ACEs & trauma informed care			
ACE acronym and study	59.7%	54.9%	0.17
Components of ACEs Training	75.8%	71.5%	0.13
Principles of trauma informed care	74.8%	70.8%	0.16

Bold values are statistically significant (P < 0.05)

Staff demonstrated improved knowledge in all domains, including knowledge of trauma, symptoms and signs of child trauma, and trauma informed care. On average staff were able to correctly identify seven of out of seven symptoms and signs of child trauma at the time of post-training assessments. In addition, nearly all staff were able to identify developmental regression (98%) and eating and sleeping problems (99%) as potential signs of child trauma at follow up. The largest improvements were noted in the ability to correctly identify the definitions of resilience and toxic stress. More than 90% of all staff correctly identified components of ACEs training, and the principles of trauma informed care at follow up (Table 2).

**Table 2:** Change in staff knowledge from baseline to follow-up (N = 1567)

	Baseline	Follow-up	P-value
Knowledge of child trauma			
Definition of trauma	99.8%	100%	0.92
Definition of resilience	47.2%	97.6%	** < 0.001**
Trauma, disease and health	98.1%	98.6%	0.89
Definition of toxic stress	67.9%	89.1%	** < 0.001**
Symptoms of child trauma	80.6%	99.1%	** < 0.001**
Trauma in early childhood	95.7%	98.7%	** < 0.001**
Knowledge of symptoms and signs of child trauma			
Avoidant	91.1%	98.4%	** < 0.001**
Anxious	95.9%	99.6%	** < 0.001**
Fearful	66.8%	99.1%	** < 0.001**
Eating and Sleeping Problems	60.9%	98.9%	** < 0.001**
Irritable	94.1%	99.4%	** < 0.001**
Loss of Skills	59.4%	97.8%	** < 0.001**
Aggressive	61.7%	98.7%	** < 0.001**
Total # of symptoms identified	5.3	6.9	** < 0.001**
Knowledge of ACEs			
ACE Acronym and Study	59.8%	96.1%	** < 0.001**
Components of ACEs Training	75.9%	92.7%	** < 0.001**
Principles of trauma informed care	74.7%	98.6%	** < 0.001**

Bold values are statistically significant (P < 0.05)

### Parents

Staff from eight agencies chose to deliver the trauma training to parents, with 443 parents completing the baseline survey. Follow up data was collected from December 2019–March 2020, during the early stages of the COVID-19 pandemic, when many agencies were closed or had limited in-person activities. One agency did not administer the follow up survey. Hence, 254 parents completed both baseline and follow up assessments across seven agencies for a follow up rate of 57%. At baseline, those lost to follow up were less likely to correctly identify the causes of trauma, less likely to identify avoiding adults as a potential symptom of trauma, less commonly reported that their child had experienced death or sudden separation, were more likely to report being unsure and less likely report being sure that their child had experienced trauma, and indicated less confidence regarding their ability to respond to child trauma. The majority of parents served by these seven participating agencies self-identified as women (85%) and white (38%), followed by Hispanic (22%), and Black (13%). The remaining groups self-identified as American Indian (7%), Asian (5%), Hawaiian/Pacific Islander (2%) and multi-racial or other (10%).

Among parents with both baseline and follow up data, at baseline, the vast majority could correctly identify potential causes of trauma with 89%–96% correctly selecting each cause. In contrast, while 85% of parents identified “avoiding adults” as a potential symptom of trauma, other symptoms were only identified by 44%–52% of parents. At baseline, nearly 56% of parents reported that child had experienced at least one adversity. However only one-third of parents believed his/her child had experienced trauma, with over a quarter unsure. In regards to attitudes, parents scored the lowest when asked whether they could help their child grow healthy and safe even if their child had experienced trauma (Table 3).

**Table 3:** Baseline statistics for parents with and without follow up data

	With follow up (N = 254)	Without follow up (N = 189)	P-value
Knowledge of child trauma			
Causes of trauma			
Being seriously hurt	95.28	85.71	** < 0.001**
Being neglected	94.49	85.19	**0.001**
Seeing someone you love hurt	95.67	84.13	** < 0.001**
Sudden separation	89.37	83.07	0.05
Number of causes identified	3.75	3.38	** < 0.001**
Responding to trauma			
Nurturing and responsive care can help a child who has experienced trauma	94.44	90.81	0.14
It's important to talk to young children about things that happened and help them understand the experience	88.00	81.28	0.05
Knowledge of symptoms and signs of child trauma			
Avoidant	85.04	76.19	**0.02**
Anxious	47.24	40.74	0.17
Fearful	48.82	47.62	0.80
Eating and Sleeping Problems	47.24	44.97	0.64
Irritable	46.06	46.03	1.00
Loss of Skills	52.36	46.03	0.19
Aggressive	44.49	40.74	0.43
Number of symptoms identified	3.71	3.42	**0.04**
Familiarity with ACEs	17.2	14.36	0.42
Attitudes			
Believes there are things can do to help your child who has experienced trauma	2.82	2.65	0.10
Knows ways to help child	2.83	2.67	**0.00**
Self-care can help child	2.75	2.61	**0.00**
Talking and listening to my child helps him/her developer relationship skills	2.59	2.43	**0.01**
Spending time together with my child (reading, singing and playing) can help him/her	2.98	2.80	** < 0.001**
I believe a mental health professional can help me if my child has experienced trauma	2.56	2.51	0.32
I can support my child by being calm and patient	2.95	2.88	**0.01**
Developing a routine can help my child feel safe and secure	2.65	2.61	0.35
Even if my child has experienced trauma, I can help him/her to grow healthy and feel safe and secure	2.14	2.19	0.52
Child trauma experiences			
Violence	29.53	29.63	0.98
Separation	27.56	19.05	**0.04**
Mental illness	30.31	24.87	0.21
Homelessness	13.78	13.23	0.87
At least 1 experience	55.91	49.74	0.20
Number of experiences	1.01	0.87	0.20
Believes child has experienced trauma			**0.03**
Yes	33.33	30.32	
No	40.16	31.38	
Not sure	26.51	38.3	

Bold values are statistically significant (P < 0.05)

At the follow up, close to 100% of parents correctly identified the causes and symptoms of trauma. In addition, many more parents (55% vs. 33%) reported their child had experienced trauma at follow up with fewer (9% vs. 27%) being unsure. Of note, there were no changes in reports of specific traumatic experiences. Parents had higher attitude scores at follow up indicating that there were more things they could do to help their child if he/she has experienced trauma, they more strongly agreed that they know ways to help their child when he/she is upset, and self-care can help their child. They also expressed stronger beliefs that specific strategies could help their child, such as talking and listening, spending time together, developing routines, and seeking help from a mental health professional. Finally, more parents agrees with the statement that, “even if my child has experienced trauma, I can help him/her grow healthy and feel safe and secure.” At baseline, 24% of parents disagreed with this statement and 39% somewhat agreed, whereas at follow up, 100% of parents strongly agreed with this statement. We observed similar patterns of improvement across all sites.

## Discussion

This virtual TTT model provided training on child trauma to over 1500 EHS/HS providers across 24 agencies in 21 states from diverse regions of the country and over 440 families across eight agencies. Staff and parents had high knowledge regarding causes of trauma at baseline. Both staff and parents, however, were better able to recognize the symptoms of child trauma following the training. Staff also had improvements in knowledge of more complex child trauma topics such as resiliency and toxic stress, and parents had an increased ability to recognize trauma in their own children. Perhaps most importantly, parents had more positive attitudes towards parenting practices known to buffer the effects of child trauma. Together, these findings suggest the program was successful in increasing knowledge and attitudes related to child trauma and parenting practices trauma (Table 4).

**Table 4:** Change in parent knowledge and attitudes from baseline to follow up (N = 254)

	Baseline	Follow-Up	P-value
Knowledge of child trauma			
Causes of trauma			
Being seriously hurt	0.95	1.00	0.076
Being neglected	**0.94**	**1.00**	**0.042**
Seeing someone you love hurt	0.96	1.00	0.094
Sudden separation	**0.89**	**1.00**	**0.042**
Number of causes identified	**3.75**	**3.99**	**0.049**
Responding to trauma			
Nurturing and responsive care can help a child who has experienced trauma	**0.94**	**0.99**	**0.031**
It's important to talk to young children about things that happened and help them understand the experience	**0.88**	**0.99**	** < 0.001**
Knowledge of symptoms and signs of child trauma			
Avoidant	**0.85**	**0.98**	**0.01**
Anxious	**0.47**	**0.99**	** < 0.001**
Fearful	**0.49**	**1.00**	** < 0.001**
Eating and sleeping problems	**0.47**	**1.00**	** < 0.001**
Irritable	**0.46**	**0.99**	** < 0.001**
Loss of skills	**0.52**	**0.99**	** < 0.001**
Aggressive	**0.44**	**0.99**	** < 0.001**
Number of symptoms identified	**3.71**	**6.93**	** < 0.001**
Familiarity with ACEs	**0.17**	**0.77**	** < 0.001**
Attitudes			
Believes there are things can do to help your child who has experienced trauma	**2.80**	**3.73**	** < 0.001**
Knows ways to help child	**2.83**	**2.93**	**0.00**
Self-care can help child	**2.75**	**2.96**	** < 0.001**
Talking and listening to my child helps him/her developer relationship skills	**2.59**	**2.96**	** < 0.001**
Spending time together with my child (reading, singing and playing) can help him/her	2.98	2.97	0.45
I believe a mental health professional can help me if my child has experienced trauma	**2.56**	**2.90**	** < 0.001**
I can support my child by being calm and patient	2.95	2.96	0.58
Developing a routine can help my child feel safe and secure	**2.65**	**2.95**	** < 0.001**
Even if my child has experienced trauma, I can help him/her to grow healthy and feel safe and secure	**2.14**	**2.96**	** < 0.001**
Child trauma experiences			
Violence	29.5%	26.0%	0.26
Separation	27.6%	30.3%	0.52
Mental illness	30.3%	24.4%	0.27
Homelessness	13.8%	15.7%	0.53
Number of experiences	1.01	0.96	0.72
Believes child has experienced trauma			
Yes	**33.33**	**55.38**	**0.003**
No	**40.16**	**35.46**	
Not sure	**26.51**	**9.16**	

Bold values are statistically significant (P < 0.05)

To our knowledge this is the first training on childhood trauma among EHS/HS providers and parents using a low-health literacy TTT approach, coupled with evaluation. A few studies have focused on the prevalence of ACEs among ECE providers, including EHS/HS providers, and their relationship to chronic health conditions (Whitaker et al., 2013, 2014). Additional studies have also explored the impact of ACEs among ECE staff on shaping classroom emotional climate for children in ECE settings (Hubel et al., 2020). This TTT approach and evaluation, however, moved beyond describing the prevalence of ACEs (i.e. types of childhood trauma) among ECE providers and focused on building capacity among ECE providers and parents of young children to understand, recognize, and respond to childhood trauma. The changes in knowledge and attitudes documented here suggest that this was accomplished, at least to some degree. In particular, the fact that fewer parents were unsure whether their child had experienced trauma at follow up as compared to baseline suggests that they were able to apply the knowledge gained to their own family. A next step would be assessing whether staff and parents, were able to change their behaviors in responding to children who have experienced trauma.

It is encouraging that staff were largely well informed regarding trauma, resilience, and trauma-informed care, even at baseline. However, important knowledge gaps were identified related to potential symptoms or signs of child trauma such as loss of developmental milestones, and eating and sleeping problems. Preschool and ECE providers are well-positioned to play a critical role in alerting parents to developmental concerns given their daily interaction, prior to school entry (Lipkin & Macias, 2020; Smith, 2020). Hence, building the capacity of the preschool and ECE workforce to recognize potential signs of trauma is a first step in helping families access the resources and support they need to help their children thrive. The importance of building workforce capacity to recognize and respond to child trauma is reinforced by the high prevalence of ACEs (Finkelhor, 2020; Whiteside-Mansell et al., 2019). Over 55% of families in this study reported at least one adverse childhood experience. This is higher than national estimates for children ages 0–5, particularly given that only a limited number of traditional ACEs were assessed (Health UDo & Services H, 2015).

Similarly, it is encouraging that parents more strongly agreed with positive parenting practices such as talking and spending time with children, providing consistent routines, and self-care as important strategies to support children who experience trauma. These practices are highly relevant and evidence-based approaches to improve social-emotional and developmental outcomes and buffer the impact of childhood trauma (Murray et al., 2019; Sullivan et al., 2016). In addition, parents were more likely to strongly agree that a mental health professional could help them if their child has experienced trauma. Increasing favorable attitudes towards mental health care may be one strategy to decrease stigma, which can be a barrier to help-seeking (Clement et al., 2014; Henderson et al., 2013).

This study is limited by the use of self-report data, which could lead to recall and social desirability bias. However, the ability to better identify socially desirable answers requires at least some knowledge of the topic. In addition, selection bias of agencies that were more motivated to learn is another limitation as participation in the training required agencies to complete an application with a written statement. Agencies were allowed to choose two of four possible mental health topics to deliver to parents so it is possible that those agencies who chose to deliver the trauma training perceived their families would be more motivated or engaged in the topic. Given that we only have baseline parent surveys from these self-selecting agencies, it is unknown whether participating parents are representative of the larger EHS/HS community. In addition, it is possible that the other mental health-related training may have reinforced the child trauma training content. Hence, it is unknown whether similar gains would be seen if the trauma training was given alone. We are also unable to assess whether changes were sustained long-term, nor whether staff and parent results varied across sociodemographic groups. Finally, although the follow up rate for staff was high, only 57% of parents who completed the trauma training and baseline assessment also completed the follow-up survey. Of note, follow up fell during the COVID 19 pandemic, which may have affected the ability of agencies to conduct assessments. Those lost to follow up appeared to have lower baseline knowledge regarding childhood trauma. On the one hand, they had greater room for improvement, though they also may have been less motivated or engaged in the topic.

Despite these limitations, our findings have important implications for parents, early childhood providers, and child health advocates. Although it appears that general awareness about childhood trauma is high, there was significant growth in knowledge and attitudes around recognizing and responding to child trauma. Hence, our results indicate a potential need to broadly disseminate similar trainings to EHS/HS parents and staff, particularly given the multiple potential risks for poor developmental and health outcomes related to trauma children and families who live in poverty may experience. Early childhood education providers are strategically poised to disseminate trauma responsive care practices as they play a critical role in children’s lives during sensitive developmental stages and as trusted community providers. Indeed, building resilience during early childhood might have long-lasting protective effects on brain development and stress regulation with the potential to mitigate many of the long-term health negative health outcomes associated with ACEs.

This low-literacy TTT approach provides a potentially promising methodology with broad dissemination potential to prepare and train the one million plus teachers and caregivers currently employed in center-based settings and the parents and families who access them. Such an undertaking is critical as knowledge and awareness of the high prevalence of child trauma, its causes, its manifestation in child behaviors, and the short- and long-term consequences of child trauma is an important first step to support practices of early recognition and responsiveness with trauma-informed care.

## Supplementary Information

Below is the link to the electronic supplementary material.

Supplementary file1 (PDF 849 kb)

Supplementary file2 (PDF 955 kb)

## Data Availability

Not applicable.

## Code Availability

All analyses were conducted in STATA. Code available upon request.
