# Supplementary material for: Improving Knowledge and Attitudes About Child Trauma Among Parents and Staff in Head Start Programs
Source: Matern Child Health J. 2022 Aug 24;26(11):2237–46. doi: 10.1007/s10995-022-03473-8 (PMC9401194; doi:10.1007/s10995-022-03473-8)
Supplement: Supplementary file 1 — Supplementary file1 (PDF 849 kb) [file 10995_2022_3473_MOESM1_ESM.pdf]

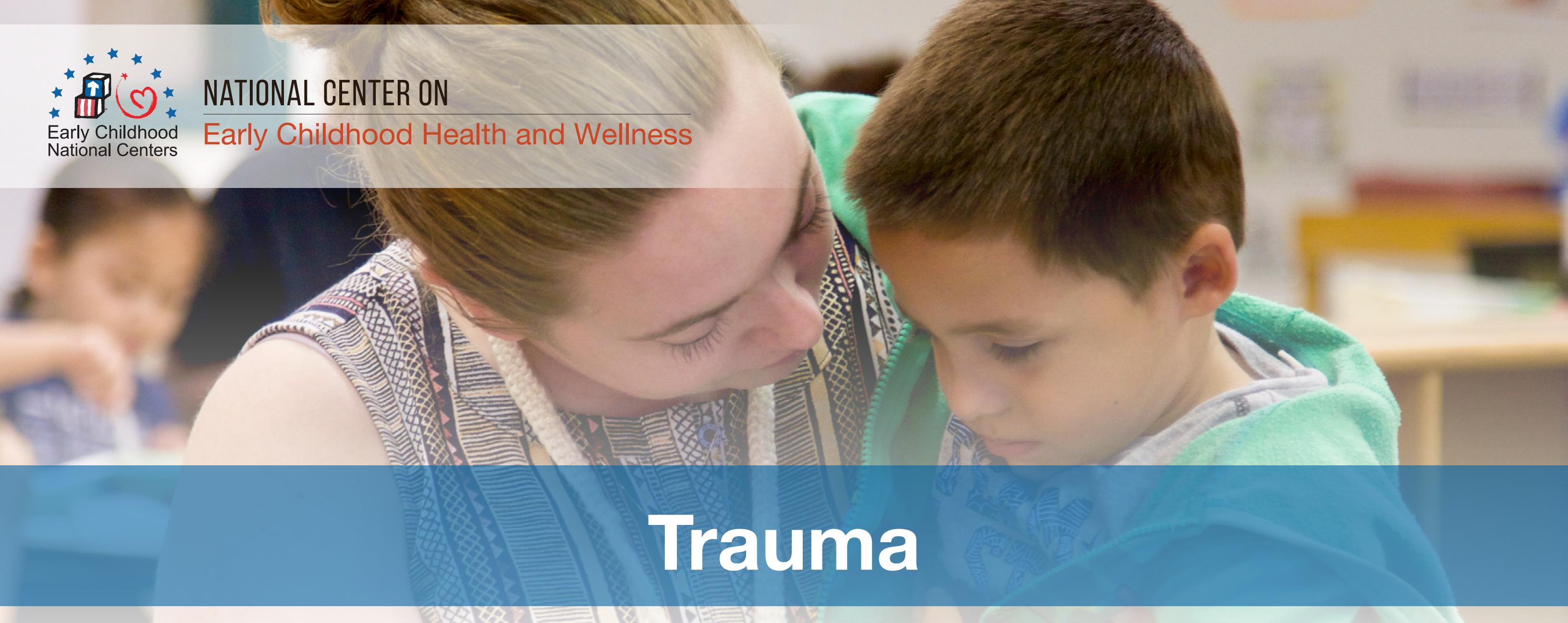

Early Childhood National Centers

NATIONAL CENTER ON
Early Childhood Health and Wellness

Trauma

Trauma: Trauma occurs when children experience an event or series of events that causes harm to their emotional or physical well-being.

Possible Traumatic Experiences:

- Being neglected
- Being seriously hurt
- Seeing a loved one be seriously hurt

Why Is Understanding Childhood Trauma Important?

- Children who have experienced trauma are often misunderstood.
- Trauma and adversity in early childhood can impact them later in life.

The **Good News** Is…

- Help is available and you can support your child to be resilient.
  - Be responsive to your child's needs
  - Try to understand the message of your child's behavior. What is your child communicating?
  - Provide reassurance that adults can help keep children safe.
  - Encourage your child to talk about his/her feelings.

Young children are always watching, listening and learning. They absorb the feelings of adults around them.

Signs and Symptoms of Trauma

In Infants & Toddlers	In Preschoolers
Problems eating	Avoid adults
Trouble sleeping	Anxious, clings to caregivers
Clingy/difficulty separating from parent	Helpless, passive
Fearful/Upset	Restless, impulsive, hyperactive
Language delay	Aggressive and/or sexually acting out behavior
Easily startled	Sadness/Irritability
Aggression	Repetitive play about the trauma
Loss of skill (e.g., stops walking even after the child is able to walk)	Poor peer relationships and social problems
	Inattention, difficulty problem solving
	Physical symptoms (e.g., headaches, etc.)
