# Supplementary material for: Improving Knowledge and Attitudes About Child Trauma Among Parents and Staff in Head Start Programs
Source: Matern Child Health J. 2022 Aug 24;26(11):2237–46. doi: 10.1007/s10995-022-03473-8 (PMC9401194; doi:10.1007/s10995-022-03473-8)
Supplement: Supplementary file 2 — Supplementary file2 (PDF 955 kb) [file 10995_2022_3473_MOESM2_ESM.pdf]

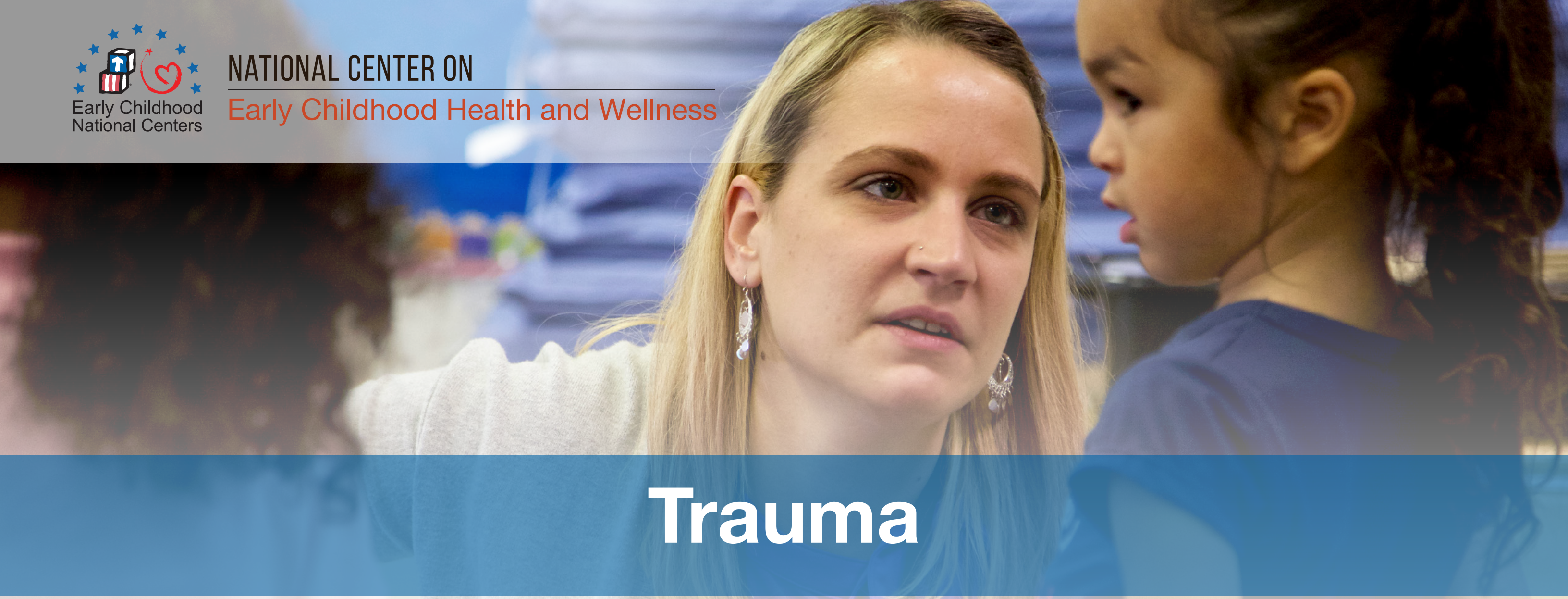

NATIONAL CENTER ON
Early Childhood Health and Wellness

Early Childhood National Centers

Trauma

Trauma:

- Children who have experienced trauma may exhibit challenging behaviors and are often misunderstood
- Children who have experienced traumatic events especially need to feel safe and loved
- If your child has been hurt, scared, or saw someone else get hurt or scared, it is important to talk to your child about what happened

Key Messages About Trauma and Young Children:

- Bad or scary experiences affect children, even very young babies
- It's important to talk to children about things that happen and help them understand the experience
- Resources are available to help children and families cope

Children Can Overcome Traumatic Experiences by Building Their Resilience.

What is Resilience?

- The ability to bounce back
- Overcoming the odds
- Recover from difficulty

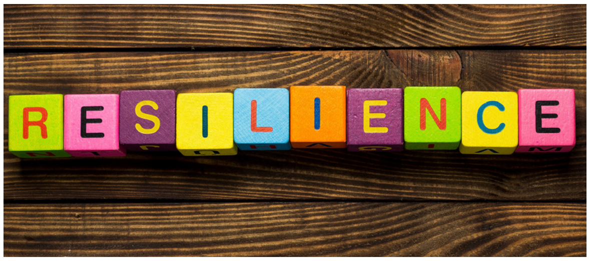

Build Your Child's Resilience

Maintain to a daily routine	Encourage children to express their feelings through play, art, and music
Provide choices	Encourage children to identify their feelings
Keep communication open with your child-listen to your child	Make time for play and creativity
Be patient	Try to understand the meaning behind your child's behavior

Take Care of Yourself (Parents):

- Take a break when you need it, if your child is safe
- Share your feelings and concerns with other adults who can listen and understand
- Talk to a mental health professional or another health professional
